# Glucose-Dependent Insulinotropic Polypeptide Prevents the Progression of Macrophage-Driven Atherosclerosis in Diabetic Apolipoprotein E-Null Mice

**DOI:** 10.1371/journal.pone.0035683

**Published:** 2012-04-20

**Authors:** Yukinori Nogi, Masaharu Nagashima, Michishige Terasaki, Kyoko Nohtomi, Takuya Watanabe, Tsutomu Hirano

**Affiliations:** 1 Department of Medicine, Division of Diabetes, Metabolism, and Endocrinology, Showa University School of Medicine, Tokyo, Japan; 2 Laboratory of Cardiovascular Medicine, University of Pharmacy and Life Sciences, Tokyo, Japan; Harvard Medical School, United States of America

## Abstract

**Aim:**

We recently reported that glucose-dependent insulinotropic polypeptide (GIP) prevents the development of atherosclerosis in apolipoprotein E-null (*Apoe* ^−/−^) mice. GIP receptors (GIPRs) are found to be severely down-regulated in diabetic animals. We examined whether GIP can exert anti-atherogenic effects in diabetes.

**Methods:**

Nondiabetic *Apoe* ^−/−^ mice, streptozotocin-induced diabetic *Apoe* ^−/−^ mice, and *db/db* mice were administered GIP (25 nmol/kg/day) or saline (vehicle) through osmotic mini-pumps for 4 weeks. The animals were assessed for aortic atherosclerosis and for oxidized low-density lipoprotein-induced foam cell formation in exudate peritoneal macrophages.

**Results:**

Diabetic *Apoe* ^−/−^ mice of 21 weeks of age exhibited more advanced atherosclerosis than nondiabetic *Apoe* ^−/−^ mice of the same age. GIP infusion in diabetic *Apoe* ^−/−^ mice increased plasma total GIP levels by 4-fold without improving plasma insulin, glucose, or lipid profiles. GIP infusion significantly suppressed macrophage-driven atherosclerotic lesions, but this effect was abolished by co-infusions with [Pro^3^]GIP, a GIPR antagonist. Foam cell formation was stimulated by 3-fold in diabetic *Apoe* ^−/−^ mice compared with their nondiabetic counterparts, but this effect was halved by GIP infusion. GIP infusion also attenuated the foam cell formation in *db/db* mice. *In vitro* treatment with GIP (1 nM) reduced foam cell formation by 15% in macrophages from diabetic *Apoe* ^−/−^ mice, and this attenuating effect was weaker than that attained by the same treatment of macrophages from nondiabetic counterparts (35%). While GIPR expression was reduced by only about a half in macrophages from diabetic mice, it was reduced much more dramatically in pancreatic islets from the same animals. Incubation with high glucose (500 mg/dl) for 9–10 days markedly reduced GIPR expression in pancreatic islet cells, but not in macrophages.

**Conclusions:**

Long-term infusion of GIP conferred significant anti-atherogenic effects in diabetic mice even though the GIPR expression in macrophages was mildly down-regulated in the diabetic state.

## Introduction

Type 2 diabetes is well known to accelerate the course of atherosclerosis, a condition associated with arterial endothelial dysfunction, macrophage foam cell formation, and vascular smooth muscle cell (VSMC) proliferation. New treatments based on incretins provide a novel approach to mediate parts of the complex pathophysiology of type 2 diabetes [1]–[2]. Incretin-based therapies have been reported to improve vascular inflammation and endothelial dysfunction beyond glucose normalization [3]. Indeed, several experimental studies have revealed that incretin-based treatments, such as glucagon-like peptide-1 (GLP-1) receptor agonists and dipeptidyl peptidase-4 (DPP-4) inhibitors, significantly suppress the development of atherosclerosis in animal models [4]–[8]. In 2011, our group reported that GLP-1 and glucose-dependent insulinotropic polypeptide (GIP), two native incretins, elicited anti-atherogenic effects in apolipoprotein-E-null (*Apoe* ^−/−^) mice, an animal model of atherosclerosis [9]. Then, more recently, we found that a DPP-4 inhibitor, an enhancer of endogenous GLP-1 and GIP, prevented the development of atherosclerosis in the same animal model [10].

It is well known that GLP-1 exerts insulinotropic action whereas GIP looses this action in diabetes [11], [12]. Several lines of experimental evidence have revealed that GIP receptors (GIPRs) in pancreatic islets are substantially down-regulated under a persistent hyperglycemic condition [13]–[17]. This may explain the inability of GIP to induce insulin secretion in diabetes. If GIPRs turn out to be down-regulated in other tissues besides the pancreatic islets, the extra-pancreatic effects of GIP will presumably be attenuated or lost. Thinking along these lines, we decided to investigate whether GIP exerts anti-atherogenic effects in diabetic *Apoe* ^−/−^ mice as well as nondiabetic *Apoe* ^−/−^ mice.

## Materials and Methods

### Animals

Animal experiments were performed in accordance with the NIH guidelines for the Care and Use of Laboratory Animals and were approved by the Institutional Animal Care and Use Committee of Showa University.

Experiment 1: Ninety 8-week-old *Apoe* ^−/−^ mice were purchased from Sankyo Labo Service (Tokyo, Japan) and kept on a normal chow until the start of the GIP infusion described below. When the *Apoe* ^−/−^ mice reached the age of 15 weeks, the animals were given multiple peritoneal injections of streptozotocin (STZ, 50 mg/kg/day) for 5 consecutive days to induce diabetes, according to the method described by Takeda *et al.* [18]. Two weeks later, animals with a 6-hour fasting blood glucose (Glutest Sensor, Sanwa Chemical, Tokyo) of greater than 200 mg/dl were selected as diabetic. These 17-week-old diabetic *Apoe* ^−/−^ mice were then infused with human GIP(1–42) (25 nmol/kg/day, Abgent, San Diego, CA) or saline (vehicle) for 4 weeks through osmotic mini-pumps (Alzet Model 1007D, Durect, Cupertino, CA) [9]. In a subset of diabetic *Apoe* ^−/−^ mice, a GIPR antagonist called [Pro^3^]GIP (25 nmol/kg/day, Abgent) was co-infused with the GIP through other mini-pumps [9]. As nondiabetic controls, 17-week-old *Apoe* ^−/−^ mice without the STZ pretreatment were infused with saline for 4 weeks through osmotic mini-pumps. When the continuous GIP infusion was commenced, the animals were also started on an atherogenic diet containing 30% fat, 20% sucrose, 8% NaCl, and 500 mg/g cholesterol (Oriental Yeast, Tokyo) [9], [19].

Experiment 2: 17 *db/db* mice, a mouse model of type 2 diabetes, and 10 *db/misty* mice, a control group, were purchased from Sankyo Labo Service at the age of 6 weeks and kept on normal chow. From the age of 8 weeks, a point at which diabetes is established to be active in *db/db* mice, 10 *db/misty* mice and 11 diabetic *db/db* mice were infused with saline (vehicle) through osmotic mini-pumps for 4 weeks, and the other 6 *db/db* mice were infused with GIP (25 nmol/kg/day) under the same conditions.

### Measurements

Four weeks after infusion, systolic blood pressure (SBP) was measured using indirect tail-cuff equipment (MK-2000, Muromachi Kikai, Tokyo) [9], [20]. Blood samples were collected after a 6-hour fast. Plasma concentrations of glucose, total-cholesterol (TC), high-density lipoprotein cholesterol (HDL-C), and triglyceride were measured by enzymatic methods using an autoanalyzer (Hitachi 7020, Hitachi, Tokyo) [20]. Non-HDL-C was calculated as TC minus HDL-C. Non-esterified fatty acid (NEFA) was measured by an NEFA C-test (Wako, Osaka, Japan). Plasma concentrations of total GIP, total GLP-1 and insulin were determined by ELISA (Millipore, Billerica, MA; Morinaga, Yokohama, Japan) [9], [10].

### Assessment of atherosclerotic lesions

Four weeks after infusion, the mice were anesthetized with diethyl ether. The whole aorta was washed with perfused PBS and fixed with 4% paraformaldehyde [20], [21]. The aorta was excised from the root to the abdominal area and the connective and adipose tissues were carefully removed. The entire aorta and cross-sections of the aortic root were stained with oil red O for the assessment of atherosclerotic lesions [20], [21]. Monocyte/macrophage infiltration into atherosclerotic lesions in the aortic roots was visualized by staining with anti-mouse MOMA-2 antibody (Abcam, Tokyo) [9], [10], [20], [21]. The atherosclerotic lesions and areas with monocyte/macrophage migration were traced by an investigator blind to the treatment and measured by an image analyzer (Adobe Photoshop, San Jose, CA and NIH Scion Image, Frederick, MD) [9], [10], [20], [21].

### Cholesterol esterification assay

The mice were given intraperitoneal injections of 4 ml of aged-autoclaved thioglycolate broth just after the 4-week infusion period, and the exudate peritoneal cells were isolated by peritoneal lavage with 8 ml of ice-cold PBS 4 days later [19], [20]. The cells were suspended in culture medium (RPMI-1640 containing 200 mg/dl glucose and supplemented with 10% FCS, 0.1 mg/ml streptomycin, and 100 U/ml penicillin) and seeded onto 6-cm dishes (4×10^6^ cells/2 ml/dish) for real-time reverse transcription polymerase chain reaction (RT-PCR) and 3.5-cm dishes (3×10^6^ cells/1 ml/dish) for cholesterol esterification assay, a standard procedure for assessing foam cell formation. After a 1-hour incubation at 37°C in 5% CO_2_ to allow adhesion, the medium was discarded to remove non-adherent cells. Adherent macrophages were incubated for 18 hours with culture medium containing 10 µg/ml human oxidized low-density lipoprotein (oxLDL) in the presence of 0.1 mM [^3^H]oleate conjugated with BSA [19], [20]. Cellular lipids were extracted and the radioactivity of the cholesterol [^3^H]oleate was determined by thin-layer chromatography [19], [20].

### *In vitro* effect of GIP on cholesterol esterification in macrophages

GIP (1 nM) was added to the cultured mediums of exudate peritoneal macrophages from nondiabetic and diabetic *Apoe* ^−/−^ mice. After 24-hour incubation with or without GIP, the cells were used for cholesterol esterification (foam cell formation) assay [9], [10], [19], [20].

### Measurements of GIPR expression

Exudate peritoneal macrophages from mice, THP-1 cells (ATCC, Manassas, VA), J774A.1 monocyte-macrophages (JCRB9108, Human Science, Osaka), and 1.2B4 pancreatic β-cells (DS Pharma Biomedical, Osaka) were suspended in culture medium and seeded onto dishes. Each type of cell was cultured under each standard culture condition and then incubated for 9 to 10 days in high-glucose medium (500 mg/dl). Total RNA was extracted from the cells using ISOGEN reagent (Nippon-Gene, Tokyo) before and after the incubation with the high-glucose medium. The cDNAs were synthesized from isolated RNA templates with a High-Capacity cDNA Archive Kit (Applied Biosystems, Carlsbad, CA). Real-time RT-PCR was performed using TaqMan Gene Expression Assays (Applied Biosystems). Pre-designed TaqMan probes for GIPR (Mm01316350_g1,Hs00164732_m1) and 18S rRNA were purchased from Applied Biosystems [9]. Amplification and fluorescent measurements were carried out during the elongation step with an ABI PRISM 7900 Sequence Detection System (Applied Biosystems).

Thin-sliced pancreases and peritoneal macrophages from mice seeded onto Chamber Slides (Iwaki Asahi Glass, Funabashi, Japan) were fixed with 4% paraformaldehyde and stained with goat polyclonal anti-GIPR antibody (Santa Cruz Biotechnology, Santa Cruz, CA). Next, the macrophages were stained with anti-goat Alexa Fluor 568 (Invitrogen, Carlsbad, CA). Nuclei and F-actin cytoskeleton were visualized using DAPI and Phalloidin Alexa Fluor 488 (Invitrogen). Fluorescence-stained cells were examined by microscope (Olympus IX71, Olympus, Tokyo).

### Statistical analysis

All values are expressed as mean ± SEM. Comparisons between 2 groups were performed by the 2-tailed unpaired Student's *t* test. Comparisons among 3 or more groups were performed by 1-way ANOVA followed by Bonferroni's post hoc test. Paired data were compared by the paired Student's *t* test. Statistics were performed using Statview-J 5.0 (SAS Institute, Cary, NC) and differences were considered statistically significant at *P*<0.05.

## Results

### Characteristics and laboratory data

Table 1 lists the blood glucose levels and other parameters in *Apoe* ^−/−^ mice. Before infusion (17 weeks of age), glucose levels were significantly higher in the vehicle-infused, GIP-infused, and GIP+[Pro^3^]GIP-infused diabetic *Apoe* ^−/−^ mice than in the nondiabetic *Apoe* ^−/−^ mice. Four weeks after infusion (21 weeks of age), glucose levels were significantly higher in the GIP-infused diabetic group than in the vehicle-infused diabetic group and GIP+[Pro^3^]GIP-infused diabetic group. Food intake in the diabetic groups was significantly higher than that in the nondiabetic group. Body weight was significantly decreased in the vehicle-infused diabetic group and improved in the GIP-infused and GIP+[Pro^3^]GIP-infused diabetic groups. There were no significant differences in the SBP or heart rate among the four groups. Plasma insulin levels in the diabetic groups were significantly lower than the plasma insulin level in the nondiabetic group. Plasma levels of TC and non-HDL-C were significantly higher in the diabetic groups than in the nondiabetic group. GIP infusion had no significant effect on TC or non-HDL-C levels. TC and non-HDL-C levels were both significantly lower in the GIP+[Pro^3^]GIP-infused diabetic group than in the vehicle- and GIP-infused diabetic groups. HDL-C levels in the diabetic groups were significantly lower than the HDL-C level in the nondiabetic group. Triglyceride and NEFA levels were both comparable among the four groups. Total GIP levels were 4-fold higher in the GIP-infused and GIP+[Pro^3^]GIP-infused diabetic groups than in the vehicle-infused diabetic group and nondiabetic group. The total GLP-1 level in each diabetic group was increased by more than 3-fold compared with that in the nondiabetic group.

**Table 1:** General characteristics and plasma measurements.

	Nondiabetic Vehicle (n = 14)	Diabetic Vehicle (n = 14)	Diabetic GIP (n = 14)	Diabetic GIP+[Pro^3^]GIP (n = 7)
Glucose (Pre-Infusion) (mg/dl)	138±9	358±24^a^	369±22^a^	368±41^a^
Glucose (Post-Infusion) (mg/dl)	104±6	246±26^a^	437±33^a^^,^ b	255±28^a^^,^ c
Food Intake (g/day)	2.5±0.2	3.8±0.3^a^	4.4±0.3^a^	4.0±0.2^a^
Body Weight (g)	31±0.6	22±1.1^a^	28±0.7^b^	29±0.7^b^
SBP (mm Hg)	108±3	106±3	108±3	109±5
Heart Rate	605±13	606±17	615±14	665±18
Insulin (ng/ml)	1.15±0.4	0.25±0.04^a^	0.27±0.03^a^	0.47±0.13^a^
TC (mg/dl)	431±35	759±88^a^	611±63	442±24^b^^,^ c
HDL-C (mg/dl)	18±1.0	13±2.4	10±0.8^a^	8±0.6^a^
Non-HDL-C (mg/dl)	413±35	746±86^a^	600±63	434±24^b^
Triglyceride (mg/dl)	90±10	52±10	88±15	34±7^a^^,^ c
NEFA (mEq/l)	1.8±0.2	2.1±0.3	1.5±0.1	1.0±0.1^b^
Total GIP (pM)	44.1±4.4	38.5±3.1	176.7±53.3^a^^,^ b	150.4±39.1^a^^,^ b
Total GLP-1 (pM)	4.4±1.4	16.1±2.8^a^	13.4±1.9^a^	15.2±2.9^a^

a = vs. Nondiabetic,

b = vs. Diabetic,

c = Diabetic GIP at *P*<0.001–0.05.

In comparison with the *db/misty* mice (n = 10), the *db/db* mice infused with vehicle (n = 11) exhibited marked hyperglycemia (371±5 vs. 110±6 mg/dl, *P*<0.0001), obesity (46±1.2 vs. 24±0.2 g, *P*<0.0001), and hyperinsulinemia (0.98±0.26 vs. 0.26±0.06 ng/ml, *P*<0.0001). In comparison with the vehicle-infused *db/db* mice, the GIP-infused *db/db* mice (n = 6) showed a significant increase in the plasma total GIP level (135±64 vs. 6.6±0.7 pM, *P*<0.05) but no change in the glucose level (374±14 vs. 371±5 mg/dl, *P* = NS).

### Atherosclerotic lesions

**Figure 1:**
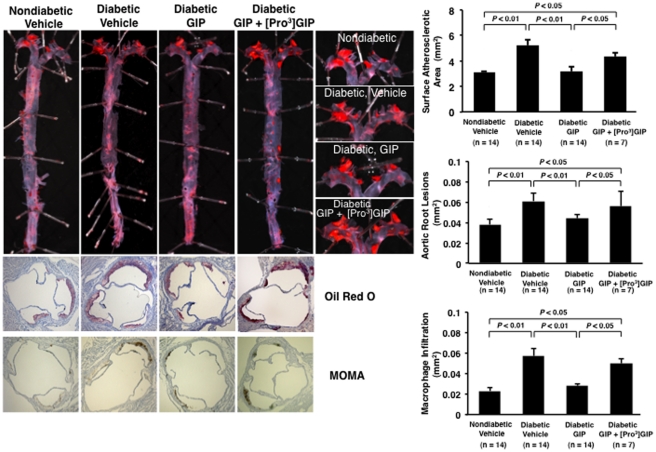
Suppressive effects of GIP on the progression of atherosclerotic lesions in diabetic *Apoe* ^−/−^ mice. Thirty-five mice at 15 weeks of age were made diabetes by peritoneal injection of STZ (50 mg/kg/day) for 5 days and 14 mice were untreated. The 17-week-old diabetic *Apoe* ^−/−^ mice were infused for 4 weeks with vehicle (control), GIP (25 nmol/kg/day), or GIP+[Pro^3^]GIP (both 25 nmol/kg/day) by osmotic mini-pumps. The aortic surface was stained with oil red O. Cross-sections of the aortic root were stained with oil red O or anti-MOMA-2 antibody. Hematoxylin was used for nuclear staining. The areas occupied by atherosclerotic lesions and by macrophage infiltration in the aortic wall were determined.

Atherosclerotic lesions were observed in 21-week-old nondiabetic *Apoe* ^−/−^ mice (Fig. 1), and further progressed atherosclerotic lesions were observed in diabetic *Apoe* ^−/−^ mice of the same age. GIP infusion remarkably reduced the surface areas of the atherosclerotic lesions and suppressed the atheromatous plaque size and macrophage infiltration in the aortic root, as compared with vehicle-infused counterparts (Fig. 1). These suppressive effects of GIP were abolished by the simultaneous infusion with [Pro^3^]GIP (Fig. 1).

The atherosclerotic lesions in the 12-week-old diabetic *db/db* mice were not remarkably developed than those in the *db/misty* mice (data not shown). When administered to the *db/db* mice, GIP failed to induce any conspicuous suppressive effects against the development of atherosclerotic lesions (data not shown).

### Foam cell formation in exudate peritoneal macrophages

**Figure 2:**
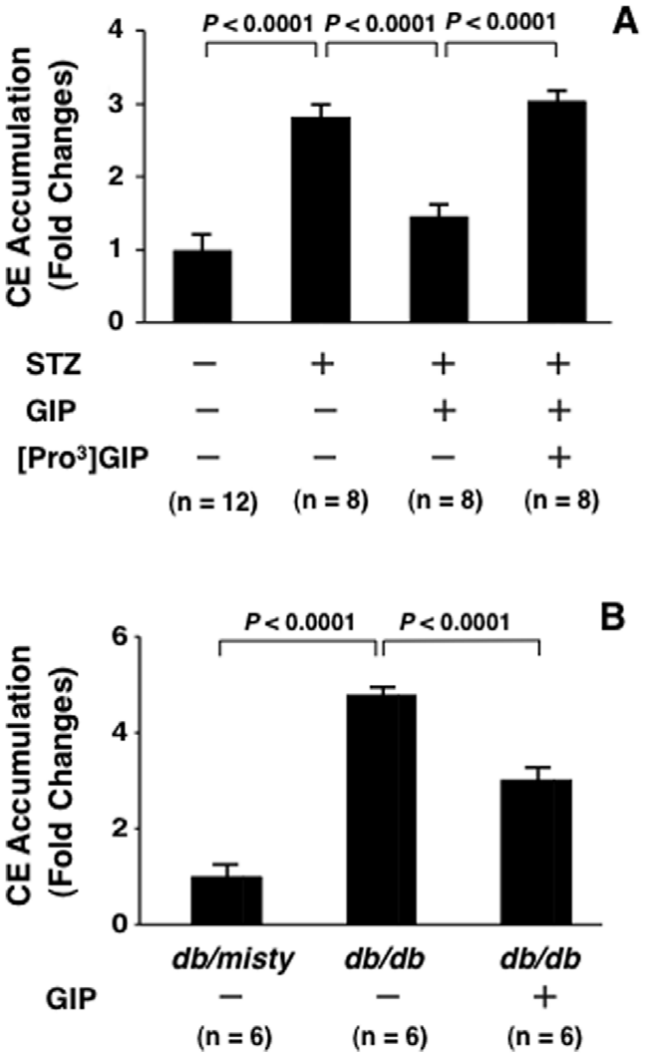
Foam cell formation in exudate peritoneal mouse macrophages. **A**, 12 *Apoe* ^−/−^ mice at 15 weeks of age were made diabetes by peritoneal injection of STZ (50 mg/kg/day) for 5 days and 6 *Apoe* ^−/−^ mice were untreated. The 17-week-old diabetic *Apoe* ^−/−^ mice were infused for 4 weeks with vehicle, GIP (25 nmol/kg/day), or GIP+[Pro^3^]GIP (both 25 nmol/kg/day) by osmotic mini-pumps. **B**, 6 *db/misty* mice and 6 *db/db* mice at 8 weeks of age were started to be infused with saline and 6 *db/db* mice were ifused with GIP (25 nmol/kg/day). Four weeks after infusions, exudate peritoneal macrophages were obtained from these mice by intraperitoneal injection of thioglycolate, and incubated with oxLDL (10 µg/ml) for 18 hours. Foam cell formation was evaluated by oxLDL-induced CE accumulation in macrophages. Assays were performed in duplicate or single. 1 fold = 17.5±1.0 nmol/mg cell protein (A) and 14.7±1.2 nmol/mg cell protein (B).

As shown in Fig. 2A, oxLDL-induced cholesterol ester (CE) accumulation in macrophages was 3-fold higher in STZ-induced diabetic *Apoe* ^−/−^ mice than in nondiabetic *Apoe* ^−/−^ mice. Foam cell formation in macrophages from GIP-infused diabetic *Apoe* ^−/−^ mice was suppressed significantly, by as much as one half, in comparison with that in the vehicle-infused counterpart. The GIP-induced suppression of foam cell formation was significantly abolished by the co-infusion with [Pro^3^]GIP.

As shown in Fig. 2B, foam cell formation was 5-fold higher in diabetic *db/db* mice than in *db/misty* mice. The GIP infusion, however, attenuated the foam cell formation significantly, by 40% in diabetic *db/db* mice.

### Expression of GIPR in exudate peritoneal macrophages and pancreatic islets

**Figure 3:**
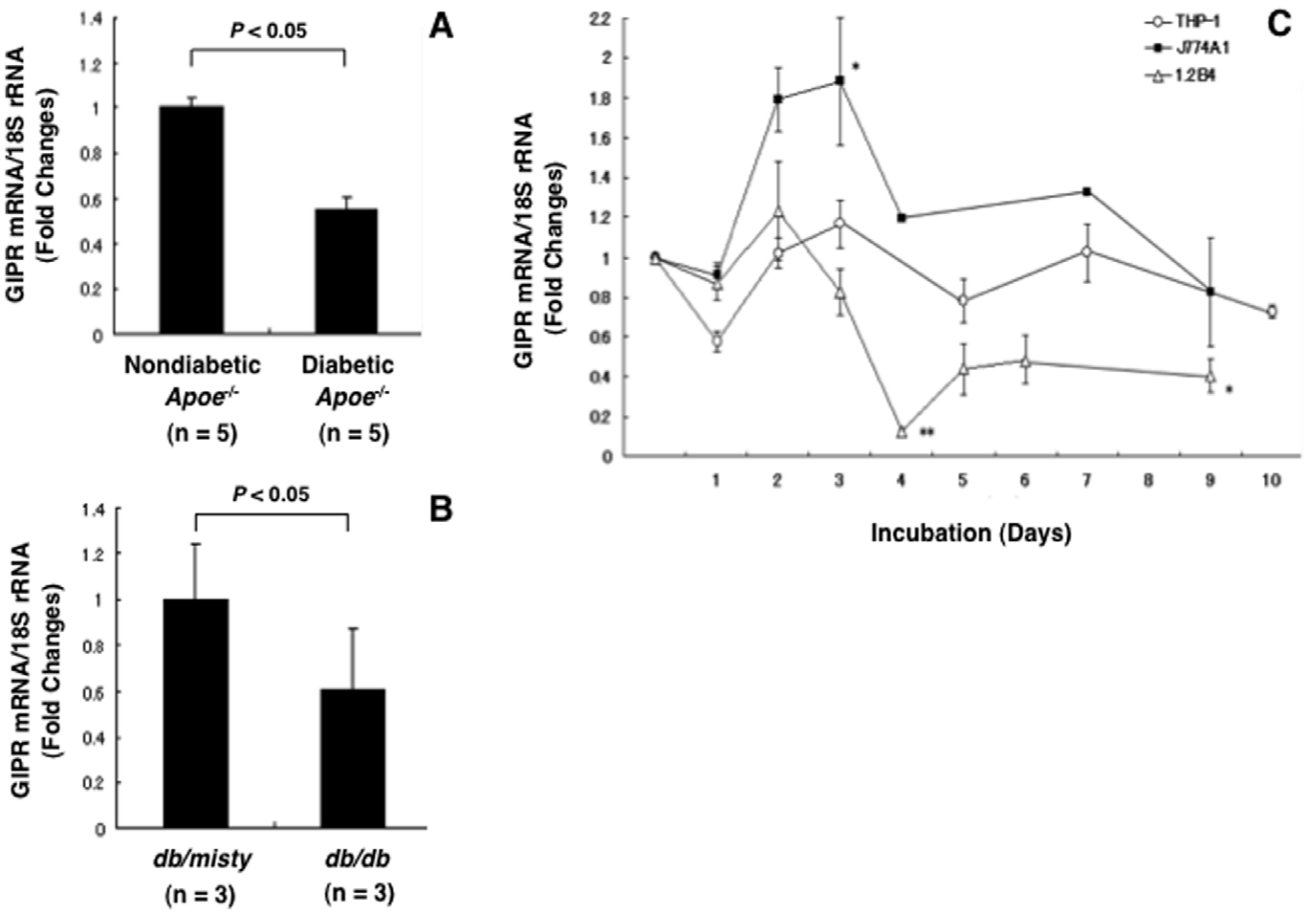
Changes in GIPR mRNA levels in macrophages and pancreatic β-cells *ex vivo* and *in vitro*. **A**, Exudate peritoneal macrophages were obtained from 5 nondiabetic mice and 5 STZ-induced diabetic *Apoe* ^−/−^ mice by intraperitoneal injection of thioglycolate. **B**, Exudate peritoneal macrophages were obtained from 3 *db/misty* mice and 3 *db/db* mice at 12 weeks of age. **C**, THP-1 macrophages (human), J774A1 macrophages (mouse), and 1.2B4 pancreatic β-cells (human) were incubated with RPMI-1640 containing high glucose (500 mg/dl) for 9–10 days. Changes in GIPR mRNA levels were determined by real-time RT-PCR. Three independent experiments were performed. *P<0.05, **P<0.01 vs. each baseline of day 0.

**Figure 4:**
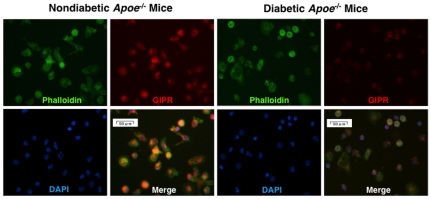
Expression of GIPR in exudate peritoneal macrophages from nondiabetic and diabetic *Apoe* ^−/−^ mice. GIPR was stained with goat polyclonal anti-GIPR antibody followed by anti-goat Alexa Fluor 568. Phalloidin/DAPI staining shows F-actin cytoskeleton and nuclear morphology of mouse macrophages. These images were merged. Representative results are shown.

**Figure 5:**
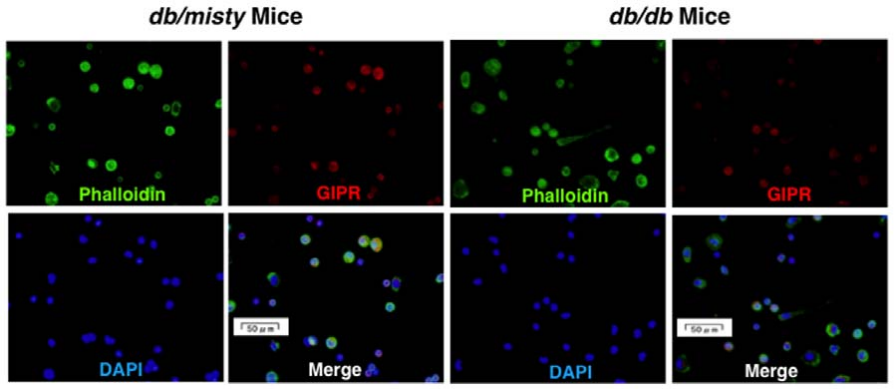
Expression of GIPR in exudate peritoneal macrophages from *db/misty* and *db/db* mice. GIPR was stained with goat polyclonal anti-GIPR antibody followed by anti-goat Alexa Fluor 568. Phalloidin/DAPI staining shows F-actin cytoskeleton and nuclear morphology of mouse macrophages. These images were merged. Representative results are shown.

**Figure 6:**
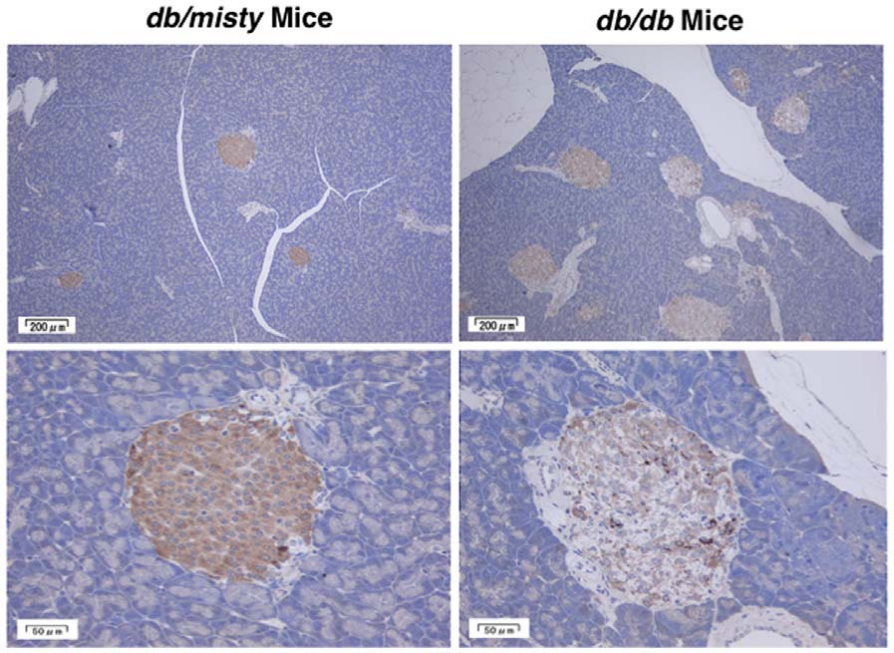
Expression of GIPR in pancreatic islets from *db/misty* and *db/db* mice. GIPR was stained with goat polyclonal anti-GIPR antibody. Hematoxylin was used for nuclear staining. Representative results are shown.

According to real-time RT-PCR, the GIPR mRNA levels in the exudate peritoneal macrophages of diabetic *Apoe* ^−/−^ mice were about half of those in nondiabetic *Apoe* ^−/−^ mice (Fig. 3A). GIPR mRNA levels in the exudate peritoneal macrophages from *db/db* mice were reduced to the same extent (Fig. 3B). Immunostaining for GIPR confirmed that the GIPR expressions were lower in the peritoneal macrophages of diabetic *Apoe* ^−/−^ mice and *db/db* mice than in those of counterpart mice (Figs. 4 and 5). Since the pancreatic islets were destroyed in STZ-treated *Apoe* ^−/−^ mice, only the islets of *db/db* mice were used for GIPR staining. The GIPR staining in the islets was much fainter in the *db/db* mice than in the *db/misty* controls (Fig. 6).

### Changes in GIPR mRNA levels in cultured macrophages and pancreatic islet cells

To determine whether the down-regulation of GIPR in diabetic animals is attributable to hyperglycemia *per se*, cultured THP-1 (human) and J774A1 (mouse) macrophages were incubated with high-glucose medium for approximately 10 days. Long-term incubation with high-glucose medium conferred no suppressive effect against GIPR mRNA levels in THP-1 macrophages or J774A1 macrophages, but it did significantly reduce GIPR mRNA levels in 1.2B4 pancreatic β-cells (human) 4 days after the start of the high-glucose incubation (Fig. 3C).

### *In vitro* effect of GIP on macrophage foam cell formation

**Figure 7:**
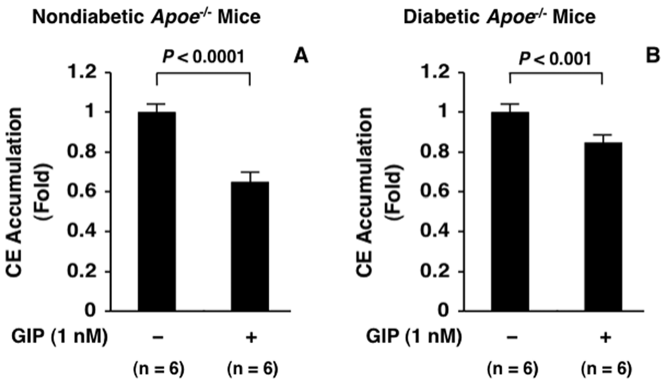
*In vitro* suppressive effects of GIP on foam cell formation in exudate peritoneal macrophages from nondiabetic and diabetic *Apoe* ^−/−^ mice. Exudate peritoneal macrophages were obtained from 6 nondiabetic *Apoe* ^−/−^ mice (A) and 6 STZ-induced diabetic *Apoe* ^−/−^ mice (B) by intraperitoneal injection of thioglycolate, and were incubated with or without GIP (1 nM) for 24 hours followed by addition of oxLDL (10 µg/ml) for 18 hours. Foam cell formation was evaluated by oxLDL-induced CE accumulation in macrophages. Assays were performed in duplicate. 1 fold = 15.6±1.5 nmol/mg cell protein (A) and 13.8±1.0 nmol/mg cell protein (B).

The 24-hour incubation with GIP (1 nM) suppressed foam cell formation significantly, by 15%, in macrophages from diabetic *Apoe* ^−/−^ mice (Fig. 7B). This was weaker than the GIP-induced suppression of foam formation in macrophages from the nondiabetic counterpart mice (35%) (Fig. 7A).

## Discussion

The atherosclerotic lesions were more severe in diabetic *Apoe* ^−/−^ mice than in nondiabetic *Apoe* ^−/−^ mice. The most prominent changes in the atherosclerotic lesions were an accumulation of macrophage foam cells in atheromatous plaques. High glucose accelerates atherosclerotic lesions and macrophage foam cell formation associated with increased uptake of oxLDL into macrophages *via* the scavenger receptor CD36 in STZ-induced diabetic *Apoe* ^−/−^ mice [22]. High glucose also down-regulates ATP-binding cassette transporters A1 and G1, key molecules for cholesterol efflux in macrophages, contributing to foam cell formation [23].

Consistent with our experience in an earlier study with nondiabetic *Apoe* ^−/−^ mice [9], our current result confirmed that chronic GIP infusion suppresses the progression of atherosclerosis in diabetic *Apoe* ^−/−^ mice. We know that GIPR mediated this anti-atherogenic effect, because co-infusion of GIPR antagonist abolished the GIP-induced suppression of atherosclerosis. GIP has a potent stimulatory effect on insulin release from the pancreas under conditions of normal glucose tolerance. Its insulinotropic action, however, is reduced or even entirely absent in a diabetic state [11], [12]. While the exact mechanisms behind the loss of GIP's insulinotropic action in diabetes remains obscure, several lines of experimental evidence suggest that GIPR is substantially down-regulated in pancreatic β-cells under a persistent hyperglycemic condition [13]–[17]. If GIPR is severely down-regulated in extrapancreatic cells in a fashion similar to pancreatic β-cells, the direct anti-atherogenic action of GIP could be largely diminished or lost. We thus anticipated that the down-regulation of GIPR may blunt the anti-atherogenic effect of GIP in diabetic animals.

GIPR gene expression in monocyte-derived macrophages from diabetic *Apoe* ^−/−^ mice and *db/db* mice, a spontaneous diabetic animal, was about half of that in nondiabetic counterparts. The *in vitro* suppressive effect of GIP on foam cell formation was blunted in macrophages from diabetic mice vs. nondiabetic mice (15% vs. 35%), probably because of the reduced GIPR in the macrophages in diabetes. As such, we were confident in surmising that the suppressive effect of GIP against atherosclerosis weakens in a diabetic condition. Yet contrary to our expectation, chronic administration of GIP *in vivo* conferred a satisfactorily anti-atherogenic effect in the diabetic *Apoe* ^−/−^ mice. The total plasma GIP concentration following subcutaneous infusion of human active GIP at a dose 25 nmol/kg/day was 130–170 pM. This was 4-times higher than the concentration in vehicle-infused mice, but not a super-physiological concentration [24]. These results suggest that GIP infusion at a level several-fold higher is sufficient to confer an anti-atherogenic effect *via* residual GIPR that eluded the diabetes-induced down-regulation. Contrary to our finding in pancreatic β-cells, high glucose left GIPR gene expression unaffected in cultured macrophages *in vitro*. We can thus infer that the down-regulation of GIPR in macrophages requires a long time, or that the suppression of GIPR expression is association with other metabolic alterations induced by diabetes. Gupta *et al.* [17], for instance, reported that peroxisome proliferator-activated receptor-γ (PPAR-γ) was reduced in the β-cells of remnant pancreas in partial pancreatectomized-diabetic rats, and that this suppression of PPAR-γ was closely associated with the down-regulation of GIPR. Plasma total GLP-1 levels were increased 3- to 4-fold in every diabetic group. Hyperphagia in STZ-induced diabetic animals may stimulate GLP-1 production in the intestines.

Glucose levels fell in our vehicle-infused diabetic mice, whereas GIP infusion worsened the hyperglycemia in diabetes. Intriguingly, co-infusion with [Pro^3^]GIP nullified GIP's pro-hyperglycemic effect. GIP infusion might exacerbate insulin resistance in the diabetic mice by increasing adiposity [25]. In contrast to our finding in STZ-diabetic mice, GIP infusion left glucose levels unchanged in spontaneous diabetic *db/db* mice. This suggests that GIP may modulate the processes of STZ-induced diabetes. The GIP infusion worsened the hyperglycemia in *Apoe* ^−/−^ mice, but atherosclerosis in these animals was milder and less developed. Meanwhile, GIPR antagonist abolished the anti-atherogenic effects of GIP in spite of its effect in reducing glucose levels. These results strongly suggest that the anti-atherogenic effects of GIP derive from mechanisms with no direct connection to the amelioration of glucose metabolism. TC and non-HDL-C levels were both higher in diabetic *Apoe* ^−/−^ mice than in nondiabetic *Apoe* ^−/−^ mice, and the HDL-C level was lower. These lipoprotein changes may accelerate atherosclerosis in diabetic mice. Plasma cholesterol levels were unaffected by GIP infusion, but they decreased when GIP+[Pro^3^]GIP was co-infused. It remains unclear why the co-infusion with [Pro^3^]GIP lowered the plasma cholesterol, but the anti-atherogenic property of GIP is probably unrelated to the improved atherogenic lipoprotein profile.

Unlike STZ-induced diabetic *Apoe* ^−/−^ mice, diabetic *db/db* mice do not develop visible atherosclerosis in the aorta. Thus, we investigated foam cell formation in exudate peritoneal macrophage as a surrogate marker for the atherosclerosis process in this animal model of diabetes. Similar to what we found in STZ-induced diabetic *Apoe* ^−/−^ mice, foam cell formation was substantially stimulated in *db/db* diabetic mice, and the GIP infusion significantly attenuated foam cell formation without affecting glucose levels. GIPR in pancreatic islets was remarkably diminished in *db/db* diabetic mice, whereas that in peritoneal macrophages was only marginally reduced. This down-regulation of GIPR in macrophages may have been limited enough to permit the suppression of foam cell formation by the several-fold increase of circulating GIP levels.

### Conclusions

Chronic administration of GIP remarkably suppressed the progression of atherosclerosis in STZ-induced diabetic *Apoe* ^−/−^ mice and suppressed macrophage foam cell formation in both diabetic *Apoe* ^−/−^ mice and diabetic *db/db* mice, even though GIPR expression in macrophages was mildly down-regulated in the diabetic state.
